# The Role of AI-Generated Clinical Image Descriptions in Enhancing Teledermatology Diagnosis: A Cross-Sectional Exploratory Study

**DOI:** 10.3390/diagnostics16030384

**Published:** 2026-01-25

**Authors:** Jonathan Shapiro, Binyamin Greenfield, Itay Cohen, Roni P. Dodiuk-Gad, Yuliya Valdman-Grinshpoun, Tamar Freud, Anna Lyakhovitsky, Ziad Khamaysi, Emily Avitan-Hersh

**Affiliations:** 1 Maccabi Healthcare Services, Tel Aviv 6812509, Israel; 2 Department of Dermatology, Rambam Health Care Campus, Haifa 3109601, Israelz_khamaysi@rambam.health.gov.il (Z.K.); e_avitan@rambam.health.gov.il (E.A.-H.); 3 Rutgers School of Public Health, Rutgers University, Piscataway, NJ 08854, USA; 4 The Bruce and Ruth Rappaport Faculty of Medicine, Technion–Israel Institute of Technology, Haifa 3525433, Israel; 5 Department of Dermatology, Emek Medical Center, Afula 18101, Israel; 6 Department of Medicine, University of Toronto, Toronto, ON M5S 3H2, Canada; 7 Department of Dermatology, Soroka Medical University Center, Ben-Gurion University of the Negev, Beer-Sheva 8410101, Israel; 8 Siaal Research Center for Family Medicine and Primary Care, Department of Family Medicine, Beer-Sheva 84105, Israel; 9 The Haim Doron Division of Community Health, Faculty of Health Sciences, Ben-Gurion University of the Negev, Beer-Sheva 84161, Israel; 10 Department of Dermatology, Sheba Medical Center, Tel HaShomer, Ramat Gan 52621, Israel; 11 Gray Faculty of Medical & Health Sciences, Tel Aviv University, Tel Aviv 6997801, Israel

**Keywords:** teledermatology, generative AI, Large Multimodal Models (LMMs), ChatGPT, automated clinical documentation, image description, artificial intelligence in medicine, diagnostic concordance

## Abstract

**Background/Objectives:** AI models such as ChatGPT-4 have shown strong performance in dermatology; however, the diagnostic value of AI-generated clinical image descriptions remains underexplored. This study assesses whether ChatGPT-4’s image descriptions can support accurate dermatologic diagnosis and evaluates their potential integration into the Electronic Medical Record (EMR) system. **Materials & Methods:** In this Exploratory cross-sectional study, we analyzed images and descriptions from teledermatology consultations conducted between December 2023 and February 2024. ChatGPT-4 generated clinical descriptions for each image, which two senior dermatologists then used to formulate differential diagnoses. Diagnoses based on ChatGPT-4’s output were compared to those derived from the original clinical notes written by teledermatologists. Concordance was categorized as Top1 (exact match), Top3 (correct within top three), Partial, or No match. **Results:** The study included 154 image descriptions from 67 male and 87 female patients, aged 0 to 93 years. ChatGPT-4 descriptions averaged 74.3 ± 33.1 words, compared to 7.9 ± 3.0 words for teledermatologists. At least one of the two dermatologists achieved a Top 3 concordance rate of 82.5% using ChatGPT-4’s descriptions and 85.3% with teledermatologist descriptions. **Conclusions:** Preliminary findings highlight the potential integration of ChatGPT-4-generated descriptions into EMRs to enhance documentation. Although AI descriptions were longer, they did not enhance diagnostic accuracy, and expert validation remained essential.

## 1. Introduction

Commercially available vision-language AI models, including ChatGPT, are increasingly integrated into daily routines and healthcare settings worldwide, assisting in medical diagnostics, education, and documentation [1,2,3]. Leveraging data from electronic medical records (EMRs), such as clinical notes, laboratory results, and billing codes, generative AI models offer enhanced predictive accuracy, simplified development (requiring fewer labeled datasets), and cost-effective deployment [4]. Generative AI has demonstrated promising applications in various medical disciplines, including radiology [4,5], otolaryngology [6], neurosurgery [7], surgical oncology [8] and dermatology [1]. Dermatology-specific multimodal systems that align image representations with clinical concepts and physician language have recently demonstrated improved diagnostic performance, highlighting the importance of domain-adapted vision language models [9]. The widespread adoption of ChatGPT, especially in medicine, necessitates a thorough exploration to define its advantages and limitations. Recent advances in artificial intelligence for dermatology reflect a broader shift from unimodal image analysis toward multimodal and clinically grounded foundation models. Early reviews highlighted the limitations of image-only systems and emphasized the importance of integrating visual data with clinical context to improve diagnostic reliability [10]. Building on this trajectory, large-scale multimodal foundation models trained specifically for dermatologic applications have recently demonstrated improved performance across multiple clinical tasks, underscoring the importance of task-specific training and structured inputs [11].

In parallel, growing attention has focused on the role of large language models within dermatologic workflows. Recent evaluations suggest that generative models such as ChatGPT may support clinical reasoning, documentation, and differential diagnosis generation, but remain insufficient as standalone diagnostic tools without structured prompting and expert oversight [12]. Together, these studies highlight the need to critically assess not only diagnostic outputs but also the quality and clinical interpretability of AI-generated documentation, particularly in teledermatology settings.

Teledermatology (TD) has become a vital healthcare delivery model, especially for patients in remote or underserved areas, by providing timely triage and convenient follow-up care [13,14,15,16,17,18,19,20,21,22]. While TD has demonstrated high diagnostic concordance with face-to-face consultations, variability in documentation quality and time constraints for physicians remain significant limitations [20,23]. An essential aspect of TD consultations is the description of clinical images—a frequently overlooked yet crucial component for ensuring clinical continuity and medico-legal accuracy [24,25]. In dermatology training, it is often taught that a high-quality clinical description is one from which the diagnosis can be easily made. This principle ensures that medical records accurately reflect the patient’s condition for continuity of care. Motivated by the global trend toward AI integration in healthcare, this study explores whether AI-generated image descriptions can meet this standard. We aimed to investigate whether an AI model can describe clinical images with sufficient clarity and relevance that a blinded dermatologist can derive an accurate diagnosis based on the description alone. AI can potentially enhance TD by supporting physicians in tasks such as analyzing and describing clinical images, integrating these analyses with metadata (e.g., demographic and clinical textual data provided by patients), and incorporating previous medical data from EMRs.

However, critical challenges remain, including risks of misinformation, algorithmic bias, difficulty interpreting unclear images, lack of direct access to chronological medical records during analysis, and limited understanding of complex clinical cases [3,26]. Validating AI algorithms is essential to confirm their accuracy, and training them requires a comprehensive and diverse dataset that encompasses a wide range of skin conditions. This validation process should involve investigating each teledermatology task separately to refine and improve it, as well as exploring the integration of data from several sources to create comprehensive consultations, make differential diagnoses, and establish recommendations.

High-quality documentation of clinical image descriptions is essential in teledermatology, not only for medico-legal purposes but also to ensure continuity of care. Leveraging AI models to assist with documentation can aid physicians by reducing documentation time and potentially identifying overlooked diagnostic details. Therefore, it is crucial to assess whether experienced dermatologists can effectively utilize AI-generated clinical descriptions to establish accurate clinical judgments. To date, limited research has specifically evaluated the diagnostic interpretability and practical utility of AI-generated image descriptions by board-certified dermatologists. Stoneham et al. demonstrated that ChatGPT-4 correctly identified primary diagnoses in 56% of cases when provided with nonspecialist-recorded histories and cutaneous descriptions, achieving an accuracy rate comparable to that of nonspecialists but significantly lower than that of dermatologists [1]. The study focused on metadata and clinical image descriptions. Mackenzie et al. reported a lower accuracy of 23% when ChatGPT-4 relied solely on clinical images sourced from DermNet™, highlighting the limitations of single-modality inputs [27]. Our recent retrospective study further demonstrated ChatGPT-4’s potential by showing a Top1 diagnostic accuracy of 87.7% when combining clinical images with metadata received via a teledermatology platform, significantly outperforming diagnoses based only on single-modality data [28].

Building upon previous work [28], this study evaluates whether diagnosticians can reach accurate diagnoses from image descriptions alone, contrasting descriptions generated by ChatGPT-4 with those initially written by teledermatologists. We aim to investigate the feasibility of integrating such descriptions into the EMR workflow in teledermatology, thus exploring a practical avenue for enhanced diagnostic accuracy and improved patient care.

## 2. Methods

This exploratory study was conducted within Maccabi Health Services (MHS), Israel’s second-largest public healthcare provider, which operates a nationwide asynchronous (store-and-forward, image-based) teledermatology service. The consultation process has been described in detail in our previous publication [28].

The teledermatologists included in this study all had at least two years of post-residency experience and also practiced in face-to-face dermatology clinics. The teledermatologists’ image description and differential diagnosis were generated in the same teledermatology session. Their diagnoses were provided as part of routine clinical assessments documented in our teledermatology service and were not independently revalidated, as the aim was to compare the investigators’ outputs with real-world teledermatologist reports. The diagnosis recorded by the original teledermatologist served as the operational reference standard for this study. We acknowledge that this represents expert clinical judgment rather than a histopathologically confirmed ‘gold standard.’ However, as these diagnoses directed actual patient care, they serve as the appropriate real-world benchmark for assessing the utility of the AI descriptions.

All teledermatologists followed written documentation guidelines, and their notes were recorded as a single free-text paragraph in a predefined sequential order. This paragraph typically included anamnesis, description of clinical images, differential diagnosis, and recommendations. The submitted images served as a surrogate for the physical examination. Dermatologists were instructed to describe the visible findings as if observed directly on the patient, specifying the location, type, and characteristics of the eruption, while explicitly noting that this constituted an image-based interpretation rather than an in-person examination. Teledermatologist documentation was written in Hebrew, while their diagnoses were recorded in English.

Although there was no rigid template, these guidelines provided a standardized framework for image description. This framework allowed the description section of the consultation to be isolated and used as the comparator against AI-generated image descriptions in this study. This exploratory study was designed to assess the feasibility, variability, and diagnostic utility of AI-generated clinical image descriptions in teledermatology. As a hypothesis-generating analysis, it aimed to identify strengths and limitations of current AI outputs and inform future studies with larger samples and predefined endpoints.

Our study included images and their descriptions from 154 teledermatology consultations conducted between December 2023 and February 2024. All images were anonymized prior to uploading to the ChatGPT-4 platform in accordance with institutional protocols, as detailed in our previous publication [28].

Patients were instructed to submit high-resolution, good-quality photographs of the affected area and were specifically advised not to use selfies or mirrors. Image sizes ranged from 313 × 312 to 1920 × 1080 pixels, consistent with our prior dataset. All analyses were performed using ChatGPT-4o. ChatGPT-4o’s data control settings were configured to prevent storage or training on study images, as previously described [28].

For each case, one image was uploaded to ChatGPT-4, which produced two distinct outputs: (1) a purely illustrative clinical image description containing no diagnostic terms and incorporating no metadata and (2) a separate differential diagnosis (DD). When a patient submitted more than one image, we preselected the highest-quality image that best represented the problem described. To avoid any carryover between cases, each consultation was initiated in a new ChatGPT session. All analyses were performed using the official OpenAI ChatGPT-4o user interface, not the API.

The generation of image descriptions was carried out using the following prompt: “Can you describe the image in a professional dermatologic way for documentation? Also, if the image is poor quality or taken through a mirror or if the dermatological condition the subject of the consultation is not sharp enough, please state so. After the description, write in one sentence only the differential diagnosis with no explanations. The differential diagnosis should be by order-most probable to least probable. If the image is blurry, a “blurry image” may be listed as the first diagnosis.”

In the current study, ChatGPT-4o and the investigators were provided with these descriptions and asked to make a differential diagnosis based only on the text. For this purpose, we created a custom GPT titled “reverse image descriptions” with the following configuration: “Reverse image descriptor is tailored for medical professionals, providing technical and concise dermatological differential diagnoses based on image descriptions. It generates a prioritized list of up to seven potential conditions, ranked from most to least probable. For descriptions mentioning that the image is blurred or not in focus, ‘blurred image’ will be listed as the number one diagnosis, acknowledging the impact of image quality on diagnosis accuracy. This feature emphasizes the tool’s precision and adaptability to the specifics of the image description, ensuring relevance and practicality for healthcare practitioners. The GPT maintains a high level of professionalism and technical specificity, making it a valuable diagnostic aid for doctors”.

The original descriptions documented by teledermatologists at the time of consultation were collected retrospectively. All descriptions provided by the teledermatologist during the teledermatology sessions, as well as those generated by ChatGPT-4, were reviewed before analysis to ensure that the descriptions themselves did not include the diagnosis within them. From the teledermatologist descriptions, eleven cases were excluded because the diagnosis was embedded in the description.

Details regarding the diagnostic categories included in the dataset, patient demographic characteristics, and the rates of concordance between AI-generated diagnoses and those of teledermatologists, based on images and metadata, have been reported in our previous study [28].

In our study, all ChatGPT-generated descriptions and teledermatologist-generated image descriptions were independently reviewed by two senior dermatologists, each with at least 10 years of post-residency experience in dermatology. These reviewers were not the same teledermatologists who initially provided the diagnoses in the teledermatology consultations. The order of case descriptions was randomized. The reviewers received only the text describing the image, and were blinded to both the clinical images themselves and the accompanying metadata. Each reviewer provided a ranked list of the three most probable diagnoses based solely on this text describing the images.

**Figure 1:**
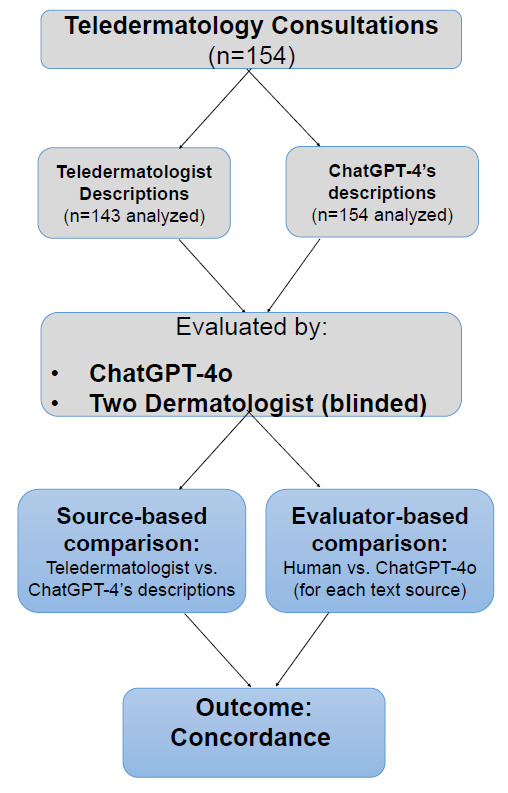
Study Design and Methodological Flow.

The descriptions were also reintroduced to ChatGPT-4o for analysis to assess its ability to derive a diagnosis based solely on generated text. In this analysis, each description was analyzed by ChatGPT-4o in a separate session, with explicit instructions that the conversation should not be saved for training. This ensured that ChatGPT-4o was not influenced by previous analyses or by the differential diagnoses generated for the images themselves. Thus, we had four groups for analysis: 154 ChatGPT-4-generated descriptions analyzed by either the investigators or by ChatGPT-4o and the 143 teledermatologists’ descriptions analyzed by either the investigators or by ChatGPT-4o. The differential diagnoses generated by each group were compared with the original diagnosis documented by the teledermatologist. Figure 1 illustrates the methodological framework.

Diagnostic concordance refers to the level of agreement between the original diagnosis made by the teledermatologist and the diagnosis made by the investigators/ChatGPT4o. It was categorized into four levels: “Top1”: The most probable diagnosis exactly matched the teledermatologist’s diagnosis. “Top3”: The correct diagnosis appeared among the top three differential diagnoses. “Partial”: A related but not identical diagnosis was included. “No”: No meaningful concordance with the teledermatologist’s diagnosis. These categories were ordered by clinical relevance, from Top1 (highest concordance) to No (lowest concordance). Since we had two investigators evaluating each description, and each achieved a different score of concordance, we applied two approaches to assess the level of agreement with the teledermatologist’s diagnosis: Maximum concordance, using the higher level of concordance provided by one of the investigators, and Minimum concordance, using the lower level of concordance between the two investigators. This approach was opposed to resolving disagreements between the two reviewers by reaching a consensus, allowing us to capture the variability and subjectivity inherent in interpreting image descriptions.

Finally, for analysis, we conducted two complementary types of analyses. The first one was “Source-based comparison” using a single evaluator: For each evaluator (ChatGPT-4o or the investigators), we compared diagnostic concordance when using ChatGPT-generated descriptions versus teledermatologist-generated descriptions. This allowed us to assess how the quality and structure of the input descriptions influenced diagnostic accuracy.

The second analysis was a “model-based comparison” using a single description source. For each set of clinical image descriptions generated by ChatGPT, we compared the diagnostic performance of different evaluators, namely, ChatGPT-4o itself and the two human investigators. For human evaluations, both the maximum concordance (the higher of the two investigators’ ratings) and the minimum concordance (the lower rating) were used.

These two analytical strategies were consistently applied across all reported concordance measures, including Top1, Top3, Partial, and No Agreement categories.

## 3. Statistical Analysis

The statistical analysis was primarily descriptive. Continuous variables were presented as means and standard deviations, while categorical variables were presented as frequencies and percentages. Diagnostic concordance rates and their corresponding 95% confidence intervals (CIs) were estimated using the Wald test, with the null hypothesis that concordance equals 50% (chance-level agreement). A *p*-value of < 0.05 was considered statistically significant. Because the analyses were exploratory and primarily descriptive, with emphasis on effect sizes and concordance percentages, no formal adjustment for multiple testing was applied; *p*-values are presented descriptively.

The sample size calculation was detailed in our previous study [28], where it was based on a hypothesized diagnostic agreement of 66–80%, drawn from a population of 7000 teledermatology consultations, with a 95% confidence level and a ±7% confidence limit.

To assess differences in description scores, McNemar’s test or the two-proportion z-test was applied. Inter-rater reliability between ChatGPT-4o and the investigators was calculated using Cohen’s Kappa, and results are reported as ranges based on minimum and maximum concordance methods. In addition, direct inter-observer concordance between the two investigators was assessed using Cohen’s Kappa.

## 4. Results

A total of 154 patients were included in the analysis. Of these, 67 (43.5%) were male and 87 (56.5%) were female. Patient age ranged from newborns to 93 years, with a mean age of 29.7 ± 19.0 years. Overall, 49 patients (31.8%) were younger than 18 years, while 105 (68.2%) were adults aged 18 years or older. ChatGPT-4 generated descriptions averaging 74.3 ± 33.1 words, compared to the teledermatologists’ average of 7.9 ± 3.0 words (*p* < 0.01).

The list of diagnoses and concordance rates is presented in Supplementary Materials File S1. The diagnostic concordance rates based on image descriptions, as compared to the diagnoses provided by teledermatologists (Source-based comparison), are summarized in Table 1, Table 2 and Table 3, and examples are presented in Supplementary Materials File S2. Analysis of the diagnostic concordance rates revealed no significant differences between the two description sources across all categories. Table 1 presents diagnostic concordance rates for ChatGPT-4o, based on both its own generated descriptions and the descriptions provided by teledermatologists. ChatGPT-4o achieved a 74.0% Top3 concordance rate when using its own descriptions and a 78.3% Top3 concordance rate when using the teledermatologists’ descriptions. Concordance rates for the investigators are presented in Table 2 and Table 3. For each diagnostic source, we report the range of concordance rates observed using the minimum and maximum concordance approaches. When basing the diagnosis on ChatGPT-4-generated descriptions, the Top 1 diagnosis concordance ranged from 28.6% (minimum approach) to 66.2% (maximum approach). The concordance rates when basing the diagnosis on the teledermatologists’ descriptions ranged from 42.0% (minimum approach to 64.3% (maximum approach).

The investigators’ Top3 diagnosis concordance with the teledermatologist’s diagnosis ranged from 54.5% (minimum approach) to 82.5% (maximum approach) when basing the diagnosis on ChatGPT-4’s descriptions and from 56.6% (minimum approach) to 85.3% (maximum approach) when basing the diagnosis on the teledermatologists’ descriptions. All *p* values are detailed in Table 1, Table 2 and Table 3.

The Cohen’s Kappa confusion matrices representing diagnostic agreement between ChatGPT-4o and investigators based on ChatGPT’s descriptions are shown in Table 4 and Table 5. Cohen’s Kappa values for agreement between ChatGPT-4o and the investigators ranged from 0.48 (the minimum approach with moderate agreement) to 0.60 (the maximum approach with substantial agreement).

The Cohen’s Kappa confusion matrices representing inter-observer concordance are presented in Table 6, demonstrating a Kappa value of 0.362, suggesting only fair concordance rates between the investigators.

Table 1, Table 2 and Table 3. Concordance Rates of Diagnoses Based on Image Descriptions Compared to Diagnoses Made by the Teledermatologist.

A: Diagnosis generated by ChatGPT-4 based on its description.
B: Maximum concordance, using the higher level of concordance provided by either investigator.
C: Minimum concordance, using the lower level of concordance between the two investigators.

Table 4 and Table 5. Confusion matrix for Cohen’s kappa, representing diagnostic agreement between ChatGPT-4 and investigators based on ChatGPT-4’s descriptions.

A: Maximum concordance, using the higher level of concordance provided by either investigator.
B: Minimum concordance, using the lower level of concordance between the two investigators.

Kappa Agreement Scale [29]:

- 0.01–0.20: Slight agreement.
- 0.21–0.40: Fair agreement.
- 0.41–0.60: Moderate agreement.
- 0.61–0.80: Substantial agreement.
- 0.81–1.00: Almost perfect (or strong) agreement.

## 5. Discussion

Artificial Intelligence (AI) has made remarkable strides in the field of image processing, particularly in its ability to generate detailed descriptions of images and even create images from textual descriptions. This capability is driven by advanced machine learning models, particularly deep neural networks, trained on vast datasets of images and corresponding descriptions.

The development of image-to-text technology has profound implications across various domains, including art [30], design [31], entertainment [32], and accessibility [33]. In teledermatology, AI can be used to rapidly generate detailed descriptions, which is crucial in dermatology where describing skin texture, color, and rashes is essential for diagnosis and follow-up. Thus, AI-generated descriptions can save time, improve the quality of medical records, and enhance the diagnostic process.

This study aimed to explore the quality and relevance of clinical image descriptions generated by ChatGPT-4 in the context of dermatology. We compared diagnostic accuracy using both AI-generated and teledermatologist-generated descriptions. Additionally, we tested the ability of highly experienced dermatologists to reach a diagnosis based on descriptions generated by either ChatGPT-4 or a teledermatologist. Notably, AI-generated descriptions were substantially longer than those written by teledermatologists. While ChatGPT-4 generated descriptions averaging 74.3 ± 33.1 words, the teledermatologists’ average was 7.9 ± 3.0 words (*p* < 0.01). Although the teledermatologists’ image descriptions were significantly shorter than those produced by ChatGPT, both investigators and ChatGPT-4o were able to reach the correct diagnosis in most cases, suggesting that concise descriptions may still convey sufficient diagnostic information when key clinical features are prioritized. Accordingly, the lengthy descriptions generated by AI did not translate into superiority in diagnostic concordance. These findings indicate that descriptive length does not automatically equate to diagnostic superiority. Studies evaluating large language models in dermatology emphasize that long or detailed descriptions do not necessarily improve diagnostic concordance unless key morphological features are clearly structured and prioritized [12]. While the expert physicians used structured descriptions and prioritized key morphological features, AI provided comprehensive details without hierarchy and treated all visible features equally. This suggests that for AI to be truly effective in clinical workflows, future prompts must be engineered to prioritize discriminatory morphological features to avoid dilution of the clinical signal.

Several studies have shown that while GPT-4-based systems may demonstrate reasonable sensitivity or alignment with evidence-based knowledge, their diagnostic precision and clinical reliability remain inferior to those of trained dermatologists or specialized models [34,35,36]. Importantly, these studies predominantly assess text-based or knowledge-driven tasks, underscoring the need for expert interpretation when applying AI-generated outputs to real-world clinical decision-making.

Dermatologists’ descriptions may be more focused, contain the essential information, and emphasize only the most clinically relevant details. Consistent with this, dermatology-specific multimodal models that explicitly align visual features with clinical concepts outperform approaches relying on unstructured narrative descriptions alone [9]. They might also include diagnostic cues and terminology that guide clinical reasoning. Notably, ChatGPT-4o also achieved the correct (Top 3) diagnosis using teledermatologists’ descriptions in nearly 80% of cases, further substantiating their accuracy. This suggests that the concise, expert-driven nature of teledermatologists’ descriptions may provide a more transparent diagnostic framework for the AI to make accurate diagnoses.

In this study, when diagnosing based on ChatGPT-4’s descriptions, at least one of the two investigators reached the exact diagnosis made by the teledermatologist in up to 66.2% of cases and within the top three in 82.5% of cases. Inter-observer concordance for diagnoses derived from AI-generated descriptions was only fair (κ = 0.362). While this suggests variability in how experienced dermatologists interpret AI-generated narratives, it also reflects the inherent limitations of free-text documentation in teledermatology. For integration into the EMR, reproducibility is as important as the richness of detail. The fair agreement we observed highlights the importance of structuring AI outputs and aligning them with documentation guidelines, ensuring that diagnostic cues are consistently emphasized. Notably, despite modest κ values, at least one of the investigators was able to achieve high Top3 concordance rates, indicating that the AI descriptions contained sufficient diagnostic information. This supports their potential implementation in EMR workflows, provided they are standardized and reviewed by teledermatologists. This suggests that AI-generated descriptions of dermatological conditions may be considered adequate. These descriptions, when reviewed and modified by the teledermatologist as necessary, could save time, enhance reproducibility, and serve as a foundation for improved machine learning through large-scale annotation.

Recent work further suggests that variability in clinician interpretation is not unique to AI-generated text but rather reflects a broader challenge inherent to free-text dermatologic documentation [12]. These findings reinforce the importance of standardized documentation frameworks and support our conclusion that AI-generated descriptions are best positioned as supportive tools within teledermatology workflows, rather than independent diagnostic agents.

Highlighting the current achievements of ChatGPT-4o in performing similarly to experienced professionals, we found that ChatGPT-4 demonstrated a substantial agreement with investigators in the “Top 3” scenario and the maximum approach when using AI-generated descriptions (Kappa = 0.6). This suggests its potential to assist in considering a broad range of differential diagnoses in clinical practice. As all investigators in this study were experienced dermatologists, results could have been different if the cases had been assessed by young senior dermatologists or family physicians/general practitioners (GPs). Moreover, while our study demonstrates the potential of AI-generated descriptions to support diagnostic reasoning, we were not able to conduct stratified analyses by lesion type, body location, or image quality due to the limited number of cases in each subgroup. Identifying patterns in AI performance across different dermatological presentations remains an important goal for future large-scale studies and could help define scenarios where AI-generated descriptions are more or less clinically reliable. Thus, for now, AI-generated descriptions are intended only as supportive documentation tools. Since the teledermatologists retain full medico-legal responsibility for validating the accuracy of the AI-generated text and the risks of fabrication, these descriptions require constant expert review, particularly in medico-legal contexts.

This study has several limitations. First, ChatGPT-4o was always provided with a single, highest-quality image per case, whereas teledermatologists at the time of consultation could review multiple images and base their description on a synthesis across them. It is possible that, in some cases, relying on and describing a single image may have disadvantaged AI. Future studies should therefore investigate ChatGPT’s potential to analyze multiple images and generate integrated descriptions. Second, the study was conducted using a single AI model (ChatGPT-4o). This model was selected as a representative of current high-performance LLMs to explore feasibility, rather than to compare models. Consequently, our results may not be directly generalizable to other generative AI systems, and future comparative studies are warranted. An additional limitation regards the use of Cohen’s Kappa to assess inter-rater agreement on diagnostic accuracy. While Cohen’s Kappa is useful for quantifying agreement, this method does not account for the type or clinical relevance of incorrect diagnoses. Cases in which both raters omitted the correct diagnosis are still counted as agreement, even if their incorrect answers differed entirely. As a result, the Kappa values presented in Table 2 may overestimate actual clinical concordance. It is also important to note that the ‘fair’ inter-observer agreement (Kappa) observed in this study likely reflects the inherent variability and subjectivity of interpreting free-text descriptions, regardless of their source. Thus, this reflects the challenging nature of text-based diagnosis rather than solely a limitation of the AI’s descriptive capabilities. Finally, this study deliberately excluded clinical metadata (e.g., age, symptoms, lesion duration) to isolate and assess the diagnostic value of the image descriptions alone. While this allowed us to evaluate the quality and sufficiency of the descriptions in supporting diagnosis, it also limits generalizability, as real-world teledermatology decisions typically integrate both visual and clinical contextual information.

In conclusion, this study demonstrates that both AI-generated and teledermatologist-generated descriptions have their strengths. While AI-generated descriptions are more detailed and can potentially save time and improve documentation, teledermatologists’ shorter, focused descriptions may be more effective in supporting accurate diagnoses. The findings suggest that while AI models can benefit from the structured, focused input of teledermatologists, human investigators excel at leveraging the comprehensive details provided by AI. Integrating AI-generated descriptions with the expert refinement of teledermatologists could offer the best of both approaches, enhancing the overall diagnostic process and documentation in teledermatology. Additionally, such findings emphasize the importance of multimodal inputs, such as combining images, clinical metadata, and structured prompts, to optimize AI performance in teledermatology.

## Figures and Tables

**Table 1:** Diagnosis generated by ChatGPT-4o based on image descriptions generated by ChatGPT-4 (left part of the table) and by the teledermatologist (right part of the table).

	ChatGPT-Generated Description			Teledermatologist-Generated Description		
ChatGPT-4 Generated Diagnosis	No./Total Cases	Concordance, % (Wald 95% CI)	*p* Value	No./Total Cases	Concordance, % (Wald 95% CI)	*p* Value
Top 1	88/154	57.1% (49.2–64.7)	0.073	91/143	63.6% (55.8–71.5)	<0.001
Top 3	114/154	74.0% (67.1–81.0)	<0.001	112/143	78.3% (71.6–85.1)	<0.001
Partial	9/154	5.8% (2.8–10.4)	<0.001	5/143	3.5% (0.5–6.5)	<0.001
No agreement	31/154	20.1% (13.8–26.5)	<0.001	26/143	18.2% (11.9–24.5)	<0.001

**Table 2:** Diagnosis generated by the investigators based on image descriptions generated by ChatGPT-4 (left part of the table) and by the teledermatologist (right part of the table). Maximum concordance, using the higher level of concordance.

	ChatGPT-Generated Description			Teledermatologist-Generated Description		
Investigator Max Concordance	No./Total Cases	Concordance, % (Wald 95% CI)	*p* Value	No./total Cases	Concordance, % (Wald 95% CI)	*p* Value
Top 1	102/154	66.2% (58.8–73.7)	<0.001	92/143	64.3% (56.5–72.2)	<0.001
Top 3	127/154	82.5% (76.5–88.5)	<0.001	122/143	85.3% (79.5–91.1)	<0.001
Partial	5/154	3.2% (0.5–6.1)	<0.001	6/143	4.2% (0.9–7.5)	<0.001
No agreement	22/154	14.3% (8.8–19.8)	<0.001	15/143	10.5% (5.5–15.5)	<0.001

**Table 3:** Diagnosis generated by the investigators based on image descriptions generated by ChatGPT-4 (left part of the table) and by the teledermatologist (right part of the table). Minimum concordance, using the lower level of concordance between the two investigators.

	ChatGPT-Generated Description			Teledermatologist-Generated Description		
Investigator Min Concordance	No./Total Cases	Concordance, % (Wald 95% CI)	*p* Value	No./Total Cases	Concordance, % (Wald 95% CI)	*p* Value
Top 1	44/154	28.6% (21.4–35.7)	<0.001	60/143	42.0% (33.9–50.1)	<0.05
Top 3	84/154	54.5% (46.7–62.4)	=0.26	81/143	56.6% (48.5–64.8)	0.1
Partial	9/154	5.8% (2.1–9.6)	<0.001	10/143	7.0% (2.8–11.2)	<0.001
No agreement	61/154	39.6% (31.9–47.3)	<0.001	52/143	36.4% (28.5–44.3)	<0.001

Note: All *p* values refer to the comparison between each group (Top 1, Top 3, Partial, and No agreement) and other diagnosis categories.

**Table 4:** Confusion matrix for Max concordance.

Kappa = 0.60, *p* < 0.001		ChatGPT-4o		Total
		Top3	None	
Investigator Max concordance	Top3	110	17	127
	None	4	23	27
Total		114	40	154

Total agreement = 133 (86.4%).

**Table 5:** Confusion matrix for Min concordance.

Kappa = 0.484, *p* < 0.001		ChatGPT-4o		Total
		Top3	None	
Investigator Min concordance	Top3	80	4	84
	None	34	36	70
Total		114	40	154

Total agreement = 116 (75.3%).

**Table 6:** Confusion matrix for inter-investigator agreement on diagnoses derived from ChatGPT-4’s generated descriptions.

Kappa = 0.362, *p* < 0.001		Second Investigator		Total
		Top3	None	
First investigator	Top3	84	13	97
	None	30	27	57
Total		114	40	154

Total agreement = 111 (72.1%).

## Data Availability

The data supporting the findings of this study are available from the corresponding author upon reasonable request, subject to any applicable privacy/ethical restrictions.

## Supplementary Materials

The following supporting information can be downloaded at https://www.mdpi.com/article/10.3390/diagnostics16030384/s1: File S1: Diagnostic Concordance Between ChatGPT and Teledermatologists. File S2: Representative Image Descriptions and Diagnostic Concordance Categories.

## Abbreviations

The following abbreviations are used in this manuscript:

AI	Artificial Intelligence
API	Application Programming Interface
CI	Confidence Interval
DD	Differential Diagnosis
EMR	Electronic Medical Record
GP	General Practitioner
GPT	Generative Pre-trained Transformer
MHS	Maccabi Health Services
TD	Teledermatology
