# Supplementary material for: The Role of AI-Generated Clinical Image Descriptions in Enhancing Teledermatology Diagnosis: A Cross-Sectional Exploratory Study
Source: Diagnostics (Basel). 2026 Jan 25;16(3):384. doi: 10.3390/diagnostics16030384 (PMC12896462; doi:10.3390/diagnostics16030384)
Supplement: Supplementary file 1 [file diagnostics-16-00384-s001.zip › Supplementary material File S1.pdf]

Supplementary material – Table S1

Table S1. Concordance between the diagnoses made by the investigators based on ChatGPT-4's generated descriptions and those given by the teledermatologists at the time of the teledermatology consultation.

Category of diagnosis	Other/Other	Top3/other	Min (Top3/Top3)	Max	Total, N
Eczema**	0 (0.0%)	4 (33.3%)	8 (66.7%)	12 (100.0%)	12
Differential diagnosis*	0 (0.0%)	6 (50.0%)	6 (50.0%)	12 (100.0%)	12
Acne / Folliculitis	1 (11.1%)	3 (33.3%)	5 (55.6%)	8 (88.9%)	9
Neoplasm not for TD	0 (0.0%)	3 (33.3%)	6 (66.7%)	9 (100.0%)	9
Viral wart	1 (16.7%)	1 (16.7%)	4 (66.7%)	5 (83.3%)	6
Alopecia	2 (33.3%)	0 (0.0%)	4 (66.7%)	4 (66.7%)	6
Onychomycosis	0 (0.0%)	0 (0.0%)	6 (100.0%)	6 (100.0%)	6
Unclear image***	3 (60.0%)	1 (20.0%)	1 (20.0%)	2 (40.0%)	5
Impetigo	1 (20.0%)	2 (40.0%)	2 (40.0%)	4 (80.0%)	5
Paronychia	0 (0.0%)	0 (0.0%)	4 (100.0%)	4 (100.0%)	4
Seborrheic dermatitis	0 (0.0%)	1 (25.0%)	3 (75.0%)	4 (100.0%)	4
Urticaria	1 (25.0%)	2 (50.0%)	1 (25.0%)	3 (75.0%)	4
Psoriasis	0 (0.0%)	0 (0.0%)	3 (100.0%)	3 (100.0%)	3
Tinea corporis	0 (0.0%)	1 (33.3%)	2 (66.7%)	3 (100.0%)	3
Tinea pedis	0 (0.0%)	0 (0.0%)	3 (100.0%)	3 (100.0%)	3
Molluscum	1 (33.3%)	2 (66.7%)	0 (0.0%)	2 (66.7%)	3
Infected wound	0 (0.0%)	2 (66.7%)	1 (33.3%)	3 (100.0%)	3
Intertrigo	1 (33.3%)	0 (0.0%)	2 (66.7%)	2 (66.7%)	3
Diaper dermatitis	0 (0.0%)	1 (33.3%)	2 (66.7%)	3 (100.0%)	3
Hemangioma	1 (50.0%)	1 (50.0%)	0 (0.0%)	1 (50.0%)	2
Dyshidrosis	1 (50.0%)	0 (0.0%)	1 (50.0%)	1 (50.0%)	2
Allergic rash	0 (0.0%)	1 (50.0%)	1 (50.0%)	2 (100.0%)	2
Tinea versicolor	0 (0.0%)	0 (0.0%)	2 (100.0%)	2 (100.0%)	2
Burn	0 (0.0%)	1 (50.0%)	1 (50.0%)	2 (100.0%)	2
Scar / keloid	0 (0.0%)	2 (100.0%)	0 (0.0%)	2 (100.0%)	2
Pityriasis rosea	0 (0.0%)	1 (50.0%)	1 (50.0%)	2 (100.0%)	2
Hematoma	1 (50.0%)	0 (0.0%)	1 (50.0%)	1 (50.0%)	2
Cutaneous leishmaniasis	2 (100.0%)	0 (0.0%)	0 (0.0%)	0 (0.0%)	2
Herpes simplex	0 (0.0%)	0 (0.0%)	2 (100.0%)	2 (100.0%)	2
Insect bite	0 (0.0%)	0 (0.0%)	2 (100.0%)	2 (100.0%)	2
Herpes zoster	1 (50.0%)	0 (0.0%)	1 (50.0%)	1 (50.0%)	2

Category of diagnosis	Other/Other	Top3/other	Min (Top3/Top3)	Max	Total, N
Hyperhidrosis	2 (100.0%)	0 (0.0%)	0 (0.0%)	0 (0.0%)	2
Infected cyst / furuncle	0 (0.0%)	0 (0.0%)	2 (100.0%)	2 (100.0%)	2
Aging of skin	0 (0.0%)	1 (100.0%)	0 (0.0%)	1 (100.0%)	1
Hand foot and mouth disease	1 (100.0%)	0 (0.0%)	0 (0.0%)	0 (0.0%)	1
Viral rash	0 (0.0%)	0 (0.0%)	1 (100.0%)	1 (100.0%)	1
Venous stasis	0 (0.0%)	1 (100.0%)	0 (0.0%)	1 (100.0%)	1
Aquagenic keratoderma	1 (100.0%)	0 (0.0%)	0 (0.0%)	0 (0.0%)	1
Autoimmune bullous disorder	0 (0.0%)	0 (0.0%)	1 (100.0%)	1 (100.0%)	1
Blepharitis	1 (100.0%)	0 (0.0%)	0 (0.0%)	0 (0.0%)	1
Tinea capitis	0 (0.0%)	0 (0.0%)	1 (100.0%)	1 (100.0%)	1
Splinter hemorrhage	0 (0.0%)	0 (0.0%)	1 (100.0%)	1 (100.0%)	1
Callus	1 (100.0%)	0 (0.0%)	0 (0.0%)	0 (0.0%)	1
Rosacea	0 (0.0%)	1 (100.0%)	0 (0.0%)	1 (100.0%)	1
Leukonychia	0 (0.0%)	1 (100.0%)	0 (0.0%)	1 (100.0%)	1
Cellulitis	0 (0.0%)	0 (0.0%)	1 (100.0%)	1 (100.0%)	1
Pitted keratolysis	1 (100.0%)	0 (0.0%)	0 (0.0%)	0 (0.0%)	1
Photodermatose	0 (0.0%)	1 (100.0%)	0 (0.0%)	1 (100.0%)	1
Granuloma annulare	0 (0.0%)	0 (0.0%)	1 (100.0%)	1 (100.0%)	1
Cyst - unspecified	0 (0.0%)	1 (100.0%)	0 (0.0%)	1 (100.0%)	1
Nothing abnormal seen	1 (100.0%)	0 (0.0%)	0 (0.0%)	0 (0.0%)	1
Ingrown hair	1 (100.0%)	0 (0.0%)	0 (0.0%)	0 (0.0%)	1
Montgomery tubercle	1 (100.0%)	0 (0.0%)	0 (0.0%)	0 (0.0%)	1
Melasma	0 (0.0%)	1 (100.0%)	0 (0.0%)	1 (100.0%)	1
Lip lickers dermatitis	0 (0.0%)	1 (100.0%)	0 (0.0%)	1 (100.0%)	1
Xanthelasma	0 (0.0%)	0 (0.0%)	1 (100.0%)	1 (100.0%)	1
	27 (17.5%)	43 (27.9%)	84 (54.5%)	127 (82.5%)	154

N- number of cases

'Top3' applied when one of the teledermatologists' diagnoses was among the investigators' most probable three diagnoses.

Other: Indicates non- or partial concordance.

Other/Other means that both reviewers showed non- or partial concordance with the teledermatologists' diagnoses.

Top3/Other means that only one of the reviewers achieved a Top-3 concordance with the teledermatologists.

Top3/Top3 means that both reviewers achieved a Top-3 concordance with the teledermatologists, corresponding to the minimum approach.

The "Max" column corresponds to the maximum approach.

* Differential Diagnosis Category - cases where the TDs was uncertain of the exact diagnosis and therefore provided a list of potential diagnoses.

** Eczema category – including atopic dermatitis, contact dermatitis, unspecified dermatitis, differential diagnosis that included only forms of dermatitis.

*** Unclear image category: Cases where the TDs requested clearer images due to poor quality.
