# Supplementary material for: The Role of AI-Generated Clinical Image Descriptions in Enhancing Teledermatology Diagnosis: A Cross-Sectional Exploratory Study
Source: Diagnostics (Basel). 2026 Jan 25;16(3):384. doi: 10.3390/diagnostics16030384 (PMC12896462; doi:10.3390/diagnostics16030384)
Supplement: Supplementary file 1 [file diagnostics-16-00384-s001.zip › Supplementaty material File S2.pdf]

Supplementary Material S2 - Example 1 - Table S2a. Representative Image Descriptions and Diagnostic Concordance Categories.

	**Teledermatologist's diagnosis - Impetigo**			
	**ChatGPT-4's image description**		**Teledermatologist description**	
	It shows a child's ear with visible skin alterations. Here are the dermatological observations: Lesion Characteristics: There is a well-demarcated, crusted lesion on the antihelix of the ear with surrounding erythema.<br><br>Erythema: There is noticeable redness surrounding the lesion, suggesting inflammation.<br><br>Crusting: There is a brownish crust atop the lesion, which may indicate a previous episode of weeping or bleeding, now dried.<br><br>Surface Texture: The lesion appears to have a rough surface texture, which is consistent with the crusting and possible excoriation.<br><br>Disruption of Skin Integrity: The lesion disrupts the normal architecture of the skin with a slight elevation, likely due to underlying inflammation or infection.		Anterior to the ear, the image shows erosions covered with crusts in a scattered distribution.	
	**Three top diagnoses based on description**	**Concordance**	**Three top diagnoses based on description**	**Concordance**
**ChatGPT-4o**	Impetigo, excoriated insect bite, traumatic excoriation	Top 1	Impetigo, Herpes Simplex, Contact Dermatitis	Top 3
**First reviewer**	atopic dermatitis, impetigo, contact dermatitis	Top 3	Impetigo, Herpes simplex, Contact dermatitis	Top 1
**Second reviewer**	Impetigo, Contact dermatitis, Seborrheic dermatitis	Top 1	impetigo, herpes simplex, herpes zoster,	Top 1

Example 2 - Table S2b. Representative Image Descriptions and Diagnostic Concordance Categories.

	**Teledermatologist's diagnosis – Hemangioma**			
	**ChatGPT-4o image description**		**Teledermatologist description**	
	The image provided shows the lateral aspect of the right upper arm with a solitary, well-demarcated, bright red macule. The lesion appears to be a small, round, flat patch on the skin, with a smooth texture and no evidence of scaling, crusting, or secondary changes such as ulceration or bleeding. The surrounding skin appears unremarkable without signs of irritation or other notable skin changes.		A purple plaque on the right shoulder.	
	**Three top diagnoses based on description**	**Concordance**	**Three top diagnoses based on description**	**Concordance**
ChatGPT-4o	Cherry angioma, spider angioma, basal cell carcinoma	Top 1	Ecchymosis, Hemangioma, Kaposi Sarcoma	Top 3
First reviewer	hemangioma, insect bite, erythema simplex	Top 1	lichen planus, ecchymosis, scar	Partial (Since list of possible diagnosis included a lesion with a vascular origin)
Second reviewer	Nummular dermatitis, contact dermatitis, atopic dermatitis	No match	Irritant contact dermatitis, allergic contact dermatitis, atopic dermatitis	No match
